# Traditional knowledge and its transmission of wild edibles used by the Naxi in Baidi Village, northwest Yunnan province

**DOI:** 10.1186/s13002-016-0082-2

**Published:** 2016-02-05

**Authors:** Yanfei Geng, Yu Zhang, Sailesh Ranjitkar, Huyin Huai, Yuhua Wang

**Affiliations:** Key Laboratory of Economic Plants and Biotechnology, Kunming Institute of Botany, Chinese Academy of Sciences, Kunming, 650201 China; University of Chinese Academy of Sciences, Beijing, 100049 China; World Agroforestry Centre East and Central Asia, Kunming, 650201 China; College of Bioscience and Biotechnology, Yangzhou University, Yangzhou, 225009 China

**Keywords:** Knowledge dynamics, Quantitative index, Organic food products, Naxi people, Gender

## Abstract

**Background:**

The collection and consumption of wild edibles is an important part in livelihood strategies throughout the world. There is an urgent need to document and safeguard the wild food knowledge, especially in remote areas. The aims of this study are to accomplish detailed investigation of wild edibles used by the Naxi in Baidi village and evaluate them to identify innovative organic food products. Also, we aim to explore the characteristics of distribution and transmission of the traditional knowledge (TK) on wild edibles among the Naxi.

**Methods:**

Data was collected through a semi-structured interview of key informants above the age of 20 years, chosen carefully by a snowball sampling. The interviews were supplemented by free lists and participatory observation methods. Informants below 20 years were interviewed to test their knowledge of traditional practices. A quantitative index like Cultural Importance Index (CI) was used to evaluate the relative importance of the different wild edibles. Linear regression and *t*-test were performed to test variation in the TK among the informants of different age groups and genders.

**Results:**

Altogether 173 wild edible plant species belonging to 76 families and 139 genera were recorded in the study. *Cardamine macrophylla*, *C. tangutorum* and *Eutrema yunnanense*, have traditionally been consumed as an important supplement to the diet, particularly during food shortages as wild vegetables. The age was found to have a significant effect on TK, but there was no significant difference between male and female informant in knowledge abundance. The traditional food knowledge was dynamic and affected by social factors. Also, it was descending partly among younger generations in Baidi.

**Conclusion:**

Baidi village is a prime example of a rapidly changing community where local traditions compete with modern ways of life. Overall, this study provides a deeper understanding of the Naxi peoples’ knowledge on wild edibles. Some wild edibles might have an interesting dietary constituent, which need in-depth studies. Such detail studies can help to promote the market in one hand and protect TK in the other. Protecting TK from disappearing in succeeding generations is necessary, and understanding the dynamics of TK is one important solution to this dilemma.

## Background

Wild plants have gained renewed interest in recent years, and the tradition of gathering wild plants continues to the present day [1, 2]. The collection and consumption of wild edibles is an important part of livelihood strategies throughout the world [3]. Wild food also is an essential supplement to the local people’s daily nutrition in developing countries [2, 4, 5]. Schunko and Vogl [6] mentioned that collection and use of wild edibles are not only part of the cultural history of a region but also are part of people’s local identity, pride, and traditions. Moreover, wild foods can contribute to overcoming periods of food scarcity, and dishes made of wild foods can be functional foods [6]. Wild plant sources and their use are under severe threat as a result of economic globalization, environmental degradation and cultural homogenization [7]. There is an urgent need to document the traditional knowledge of plant uses and conserve its habitat [7–9], especially where it is not yet completely lost [10]. Wild edibles are not an exception to this fact. It is important to document local knowledge before it vanishes along with the knowledgeable people, in the sense that it is slowly disappearing with the demise of those who have traditionally upheld it [11].

China is a fascinating and significant arena for studies on wild food use traditions, particularly Yunnan province [12]. Northwest Yunnan is one of biodiversity hotspots and is home to many minority groups. Some ethnobotanical researchers have documented wild edibles used by different minorities of this region [13–18].

**Fig. 1:**
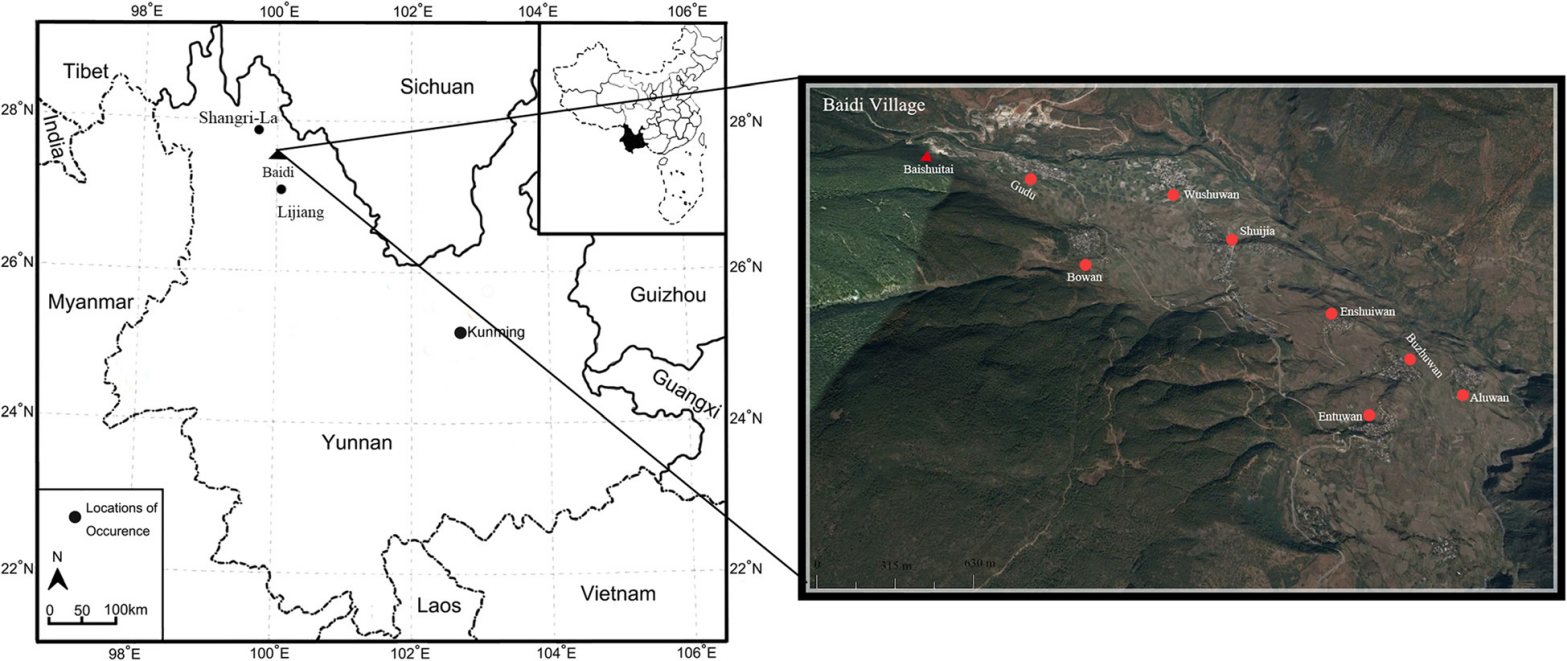
The location of Baidi village and its small groups

The Naxi people, one of the main ethnic groups in northwest Yunnan, have accumulated rich knowledge on using wild edibles. Baidi Village (Sanba Naxi Nationality Township, Shangri-La City, Deqing Prefecture) is located in 27° 30′ N to 27° 28′ N and 100° 01′ E to 100° 05′ E, the Northwest of Yunnan Province, roughly between the two cities Lijiang and Diqing (Fig. 1). It is 103 kilometers from Shangri-La City and 170 kilometers from Lijiang city. The mountain in its territory belongs to Haba Snow Mountain, Yunling Mountain range. Baidi has an area of 8.26 km^2^ and reaches an elevation of approximately 4500 m while networks of streams and rivers including Geji and Yangtze dissect numerous valleys, which make it encompass a rich diversity of plants. The village has 15 sections or groups of the settlement, eight of which belong to the Naxi (Fig. 1). In the northwest of the village, there is a big limestone terrace, Baishuitai (literal meaning white water terrace). Local people believe this place as a shrine and perform various religious activities [19]. It also is a famous scenic spot that attracts the considerable number of tourists all over the world.

Baidi comprises approximately 3000 inhabitants, and the majority of them are the Naxi ethnic minority along with about 25 % of the Han people and the Yi people. The Naxi in Baidi is culturally related to the Lijiang Naxi, but they are usually considered the purest of their race [20, 21]. Joseph Rock, who is a well-known researcher, studied the Naxi people closely and mentioned that the Naxi in Baidi is the most aboriginal among Naxi, and they follow their old religious customs, which are a mixture of shamanism and the pre-Buddhistic Bon religion of Tibet. There are neither Lama temples nor Chinese temples as in the Lijiang city. The Naxi believes that mountains, rivers, trees, herbs, animals and humans, all have their unique spirits. Among these spirits of nature, the Shu spirits are the most important. According to a Naxi myth, farmland and livestock are in the realm of men while Shu rules the mountains and the rivers. Men frequently invaded the territory of Shu creating hostility and fights between men and Shu. Dongba priests, the mediators with spiritual powers, were then called to regain the harmony between them. They agreed that human beings must worship the Shu god of nature every year, in return Shu would provide men’s need from nature and stop assaulting them. In this way, men and Shu lived in harmony afterward [21]. The religion and ceremonies of the Naxi represent the long history of keeping equilibrium between man and nature to guarantee the sustainability of natural resources.

Wild edibles in this article refer to those plants that grow without cultivation, including fungi and lichen, and consumed by Naxi people or local animals. It mostly includes native species growing in their natural habitat, but sometimes managed, as well as introduced species that have been naturalized [22]. In this paper, we documented angiosperm, gymnosperm, fern, fungi, lichen and algae, which are sources of vegetables, vitamin and functional food, forage, starch and sugar, edible pigments, oil and fats, beverage and honey source.

This study aims to accomplish detailed investigation into wild edibles used by the Naxi in Baidi village and evaluate them to identify innovative organic food products. Also, we aim to explore the distribution of traditional knowledge (TK) and its transmission pathways to the young generation of Naxi.

## Methods

### Data collection

The fieldwork was conducted in 2013 and 2014. Field studies included free lists, semi-structured interviews, and participatory observation. The total of 86 key informants was selected using snowball sampling [23, 24]. The ages of informants ranged from 21 to 91 (mean age 57 years old), and the sex ratio of informants was almost 1:1 (male to female was 42 to 44). To that 20 other participants below an age of 20 years (mean age 14 years old) were randomly invited. These youngsters were asked to fill the questionnaire with the purpose of documenting the traditional knowledge transmission.

In the first phase of the field research, participants were invited to list all wild edibles still used on a regular basis, and those were used only in the past. The interviews include the questions that were relevant to document detail information on all wild edibles including the source of knowledge about plant use. Every use report on edible plants included (1) number of useful plants mentioned and their botanical families, (2) most frequently used plant parts, (3) most cited species, (4) ways of consumption and preparation, (5) season of collection, (6) habitats where collected. In the second phase, we collected the wild edibles mentioned above with local gatherers. The participatory observation was utilized to secure the cultural implication of plant gathering, preparation, and distribution of wild edibles. Nomenclature of all vascular plants follows *Flora of China* [25], and the voucher specimens deposited at the herbarium of the Kunming Institute of Botany, CAS (KUN).

### Data analysis

Ethnobotanical information collected from 86 key informants was properly documented and analyzed. We classified the wild edibles into the following categories based on usage or main chemical composition: carbohydrates, protein, oil and fats, vegetable, vitamin and functional food, beverage, condiments, forage, honey source and chewing and stimulate plants.

To quantify the use frequency of certain species, we calculated the utilization frequency [26], using following formula:

$$ f=\frac{{\mathrm{N}}_m}{{\mathrm{N}}_i} $$

In this formula, *f r*epresents the utilization frequency, N_m_ is the number of informants mentioned certain species, N_i_ represents the total number of informants. Higher the value of *f*, the more frequent is the plant used.

Each species mentioned by an informant within one food category was a use report (UR). To determine diversity of uses and the consensus of informants, we used the Cultural Importance Index (CI), which can be mathematically expressed as [27]:

$$ {\mathrm{CI}}_{\mathrm{s}}={\displaystyle \sum_{\mathrm{u}={\mathrm{u}}_1}^{{\mathrm{u}}_{\mathrm{N}\mathrm{C}}}}{\displaystyle \sum_{\mathrm{i}={\mathrm{i}}_1}^{{\mathrm{i}}_{\mathrm{N}}}}{\mathrm{UR}}_{\mathrm{u}\mathrm{i}/\mathrm{N}} $$

N is the total number of informants, and NC is the total number of use categories. Therefore, the CI is the sum of the proportion of informants that mention each of the use categories for a given species. This index indicates the spread of the use (number of informants) of each species, as well as the diversity of its uses. Every additional use category is a measure of the relative importance of each plant use [27]. Therefore, multiple uses of a species is an indicator of higher CI value.

Also, the Cultural Food Significance Index (CFSI) was calculated to evaluate the cultural significance of wild edibles using following formula given by Andrea Pieroni [28]:

$$ \mathrm{CFSI}=\mathrm{QI}\times \mathrm{AI}\times \mathrm{F}\mathrm{U}\mathrm{I}\times \mathrm{P}\mathrm{U}\mathrm{I}\times \mathrm{MFFI}\times \mathrm{TSAI}\times \mathrm{F}\mathrm{MRI}\times {10}^{-2} $$

This index takes into consideration a wide variety of factors in the evaluation of a specific wild edible. The CFSI include quotation frequency (QI, frequency of quotation index), availability(AI, availability index), typology of the used parts(PUI, parts used index), frequency of use (FUI, frequency of utilization index), kind and number of the food uses (MFFI, multifunctional food use index), taste appreciation (TSAI, taste score appreciation index) and perceived role as food medicine (FMRI, food-medicinal role index). The use of this index allows for exploring the potential wild greens.

To analyze how TK varied according to the characteristics of the different informants, we performed linear regression and *t*-test using R software (version 3.2.2), taking “Number of edible plants cited by each informant” as the variable to the model. We also consider two entities representing personal data, “ages” (a quantitative variable) and “gender” (a qualitative variable taking a value of male or female). Furthermore, documentation of our field investigation was compared with the nutrition information reported in the various relevant literatures.

## Results and discussion

**Fig. 2:**
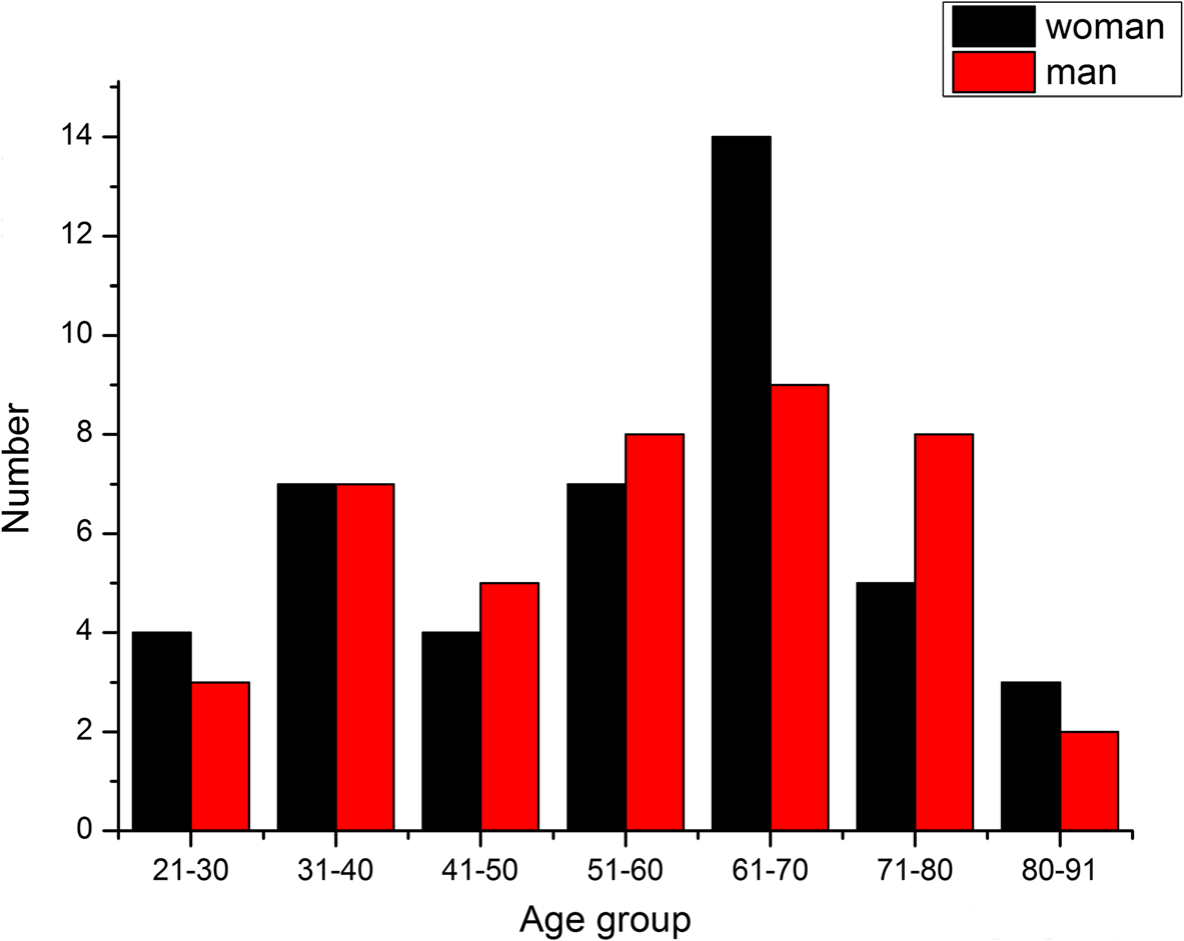
The age structure of 86 key informants

The traditional diet culture of the Baidi village has developed from nomadic lifestyle into an agricultural and pastoral context. Cultivated species play a crucial role in the local diet, but they have a long history of wild edibles gathering. The 86 informants (Fig. 2) of Baidi village reported 173 wild edible species belonging to 76 families and 139 genera (Table 1) that they still collecting or had gathered in the past. Table 1 lists the wild edibles mentioned at least by two informants. Botanical and ethnobotanical information about these plants include scientific name, family, voucher or digital photograph number, vernacular name, food categories, part(s) used and mode of consumption (prevalence of use) and collecting habitat (season) [29]. Food categories include carbohydrates, oil and fats, vegetable, vitamin and functional food, beverage, condiments, forage, and honey source. On average, 20.6 edible taxa were listed per informant. The highest number of wild edibles included vegetables (mean – 13.2 species), whereas vitamins and functional foods were frequently used (mean – 7.4). Other categories were less frequent in use such as carbohydrates (mean –0.4), Edible pigments (mean –0.36), Oil and fats (mean – 1.8), Beverage (mean –0.34), Honey source plant (mean –0.23). CI and CFSI values of the wild edibles, except the forage category, cited at least three times were calculated (Table 1).

**Table 1:** Inventory of wild edibles gathered and consumed in the Baidi village

Taxon	Family	Vernacular name	Food categories	Part(s) used and mode of consumption (prevalence of use^a^)	Collecting habitat^b^ (season)	Voucher number	FC	f	CI	CFSI
Angiosperma										
*Acorus gramineus* Sol. ex Aiton	Acoraceae		vitamine & functional food	Rhizomes, boiled in water without garnish (TC).	AE(all seasons)	P1408				
*Amaranthus* sp.	Amaranthaceae		vegetable	Leaves, fried (TC).	SC-CA-UA (spring)	0354				
*Chenopodium album* L.	Amaranthaceae	mulv	vegetable	Leaves, fried (TC).	CA-UA (spring and summer)	0151				
*Kochia scoparia* (L.) Schrad.	Amaranthaceae		vegetable	Leaves, fried (TC).	CA-UA (spring and summer)	P1413				
*Allium* sp.	Amaryllidaceae	gu	edible condiments, vegetable	Leaves, fried (TC).	FO (spring and summer)	0355	40	0.47	0.47	24.00
*Pistacia weinmanniifolia* J. Poiss. ex Franch.	Anacardiaceae	yizhu	vitamine & functional food	Fruits, eaten raw (AB).	FO-CA-UA(summer and autumn)	0055	16	0.19	0.19	5.40
*Ligusticum sinense* cv. *Chuanxiong* S. H. Qiu & et al.	Apiaceae		vitamine & functional food	Roots, boiled in water (TC).	FO(all seasons)					
*Oenanthe javanica* (Bl.) DC.	Apiaceae	zen axi	vegetable	Leaves, fried (TC).	AE(all seasons)	0045	31	0.36	0.36	27.90
*Cynanchum auriculatum* Royle ex Wight	Apocynaceae	niezi	vegetable	Leaves and stems, boiled in water (AB).	SC-UA (all seasons)	0088				
*Marsdenia* sp.	Apocynaceae	Lubei	vegetable	Leaves and stems, boiled in water (AB).	SC (spring and summer)	0234				
*Amorphophallus konjac* K. Koch	Araceae	Bulei	carbohydrates	Tubers, dried, smashed and boiled in water for making curd (TC).	FO-CA-SC (autumn)	0052				
*Arisaema elephas* Buchet	Araceae	Babaxiluo	forage, vitamine & functional food	Roots, boiled in water (TC). Leaves, eaten raw as forage (TC).	SC-CA-UA (all seasons)	0048				
*Arisaema erubescens* (Wall.) Schott	Araceae	Rihaxiluo	forage, vitamine & functional food	Roots, boiled in water (TC). Leaves, eaten raw as forage (TC).	SC-CA-UA (all seasons)	0095				
*Asparagus cochinchinensis* (Lour.) Merr.	Asparagaceae	Laosha	vitamine & functional food	Roots, boiled in water (TC).	FO-SC-UA (all seasons)	0047				
*Maianthemum japonicum* (A. Gray) La Frankie	Asparagaceae	Abu	vegetable	Leaves, fried (TC).	FO (spring and summer)	0011	53	0.62	0.62	55.65
*Arctium lappa* L.	Asteraceae	Elaba	vegetable, vitamine & functional food	Roots, stewed (TC).	SC-CA-U A (all seasons)	0258	4	0.05	0.05	7.02
*Artemisia sieversiana* Ehrhart ex Willd.	Asteraceae		forage, vitamine & functional food	Whole plant, boiled in water (AB). Aerial part, eaten raw as forage (TC).	FO-SC-CA-UA (spring, summer and autumn)	0137				
*Carpesium cernuum* L.	Asteraceae	La men ga	forage, vitamine & functional food	Whole plant, boiled in water (TC). Aerial part, eaten raw as forage (TC).	SC-UA (all seasons)	0299				
*Carpesium* sp.	Asteraceae	La men ga	forage, vitamine & functional food	Whole plant, boiled in water (TC). Aerial part, eaten raw as forage (TC).	SC-UA (all seasons)	0150				
*Cichorium intybus* L.	Asteraceae		vegetable, forage	Leaves, fried (TC).	SC-CA-UA (all seasons)	P1407				
*Cirsium lidjiangense* Petr. & Hand.-Mazz.	Asteraceae	Raqiku	vegetable, vitamine & functional food	Roots, stewed (TC).	SC-CA-UA (all seasons)	0260	3	0.03	0.03	5.27
*Galinsoga parviflora* Cav.	Asteraceae	Munukepei; Youcong	forage	Aerial part, eaten raw (TC).	CA (spring, summer, autumn)	0020				
*Hippolytia delavayi* (Franch. ex W. W. Smith) C. Shih	Asteraceae	Bunasi	vitamine & functional food	Roots, boiled in water (TC).	FO (all seasons)	0114				
*Leibnitzia anandria* (L.) Turcz.	Asteraceae	Mumeicidei	forage	Aerial part, eaten raw or boiled in water (AB).	CA (spring, summer, autumn)	0061				
*Sigesbeckia orientalis* L.	Asteraceae	Umeiheiba	forage	Aerial part, eaten raw or boiled in water (TC).	CA (spring, summer, autumn)	0101				
*Sonchus oleraceus* L.	Asteraceae	Umeisennier	vegetable	Leaves, fried (TC).	CA-UA (spring)	P1420				
*Taraxacum mongolicum* Hand.-Mazz.	Asteraceae	Pugongying	vegetable, vitamine & functional food	Whole plant, boiled in water (TC).	SC-CA-UA (all seasons)	0189	70	0.81	0.85	157.50
*Begonia grandis* Dryand.	Begoniaceae	Akangzi	vegetable	Tender leaves and stems, eaten raw (AB).	FO-CA-UA(summer and autumn)	0087				
*Berberis* sp.	Berberidaceae	Ciilv	vitamine & functional food	Fruits, eaten raw (TC).	FO-CA-UA(summer and autumn)	0007	4	0.05	0.05	1.35
*Cynoglossum amabile* Stapf & J. R. Drumm.	Boraginaceae		forage	Aerial part, eaten raw or boiled in water (AB).	CA(spring, summer, autumn)	0064				
*Ehretia dicksonii* Hance	Boraginaceae	Buna	forage, vitamine & functional food	Fruits, eaten raw (AB). Leaves, as forage (AB).	SC-UA (summer)	0207	4	0.05	0.05	1.35
*Capsella bursa-pastoris* (L.) Medik*.*	Brassicaceae		vegetable	Leaves, fried (TC).	SC-CA-UA (spring)	0198				
*Cardamine macrophylla* Willd.	Brassicaceae	You	vegetable	Leaves, fried (TC).	FO (spring and summer)	0266	76	0.88	0.94	205.20
*Cardamine tangutorum* O. E. Schulz	Brassicaceae	You	vegetable	Leaves, fried (TC).	FO(spring and summer)	0353	76	0.88	0.94	205.20
*Eutrema yunnanense* Franch.	Brassicaceae	Bei	vegetable, forage	Leaves, fried (TC). Eaten raw by animals.	FO (spring and summer)	0352	73	0.85	0.85	65.70
*Nasturtium officinale* R. Br.	Brassicaceae	Shuicai, Xiyangcai	vegetable	Leaves, fried (CC).	AE(all seasons)	0166	45	0.52	0.52	206.72
*Thlaspi arvense* L.	Brassicaceae	Jucu	oil & fats, vitamine & functional food	Seeds, dried and boiled in water (AB). Whole plant, boiled in water as functional food (AB).	SC-UA (summer)	0129				
*Adenophora stricta* Miq.	Campanulaceae	Apudada	vitamine & functional food, vegetable	Roots, stewed in meat (TC). Leaves, eaten raw (TC).	CA(all seasons)	0038	38	0.44	0.45	106.88
*Cannabis sativa* L.	Cannabaceae	Samei	oil & fats	Seeds, dried and boiled in water (AB).	SC-CA-UA (summer and autumn)	P1422	67	0.78	0.78	24.12
*Dipsacus asper* Wall. ex DC.	Caprifoliaceae		vitamine & functional food	Roots, boiled in water (TC).	FO-SC-CA-UA(all seasons)	P1421				
*Sambucus adnata* Wall. ex DC.	Caprifoliaceae	Shousi	vitamine & functional food	Whole plant, boiled in water (TC).	FO-SC(all seasons)		3	0.03	0.03	1.01
*Sambucus javanica* Blume	Caprifoliaceae	Munongzi	vitamine & functional food	Whole plant, boiled in water (TC).	SC-CA-UA (all seasons)	0227				
*Valeriana jatamansi* Jones	Caprifoliaceae	Matixiang	vegetable, vitamine & functional food	Whole plant, stewed (TC).	SC-CA-UA (all seasons)	0041	67	0.78	0.90	120.60
*Viburnum betulifolium* Batalin	Adoxaceae	Efuni	vitamine & functional food	Fruits, eaten raw (TC).	FO-CA-UA(summer and autumn)	0122	30	0.35	0.35	12.15
*Viburnum cylindricum* Buch.-Ham. ex D. Don	Adoxaceae		oil & fats	Seeds, dried and boiled in water (AB).	FO-SC (winter)	0035				
*Viburnum foetidum var. ceanothoides* (C. H. Wright) Handel-Mazzetti	Adoxaceae	Ciifuni	vitamine & functional food	Fruits, eaten raw (TC).	FO-CA-UA(summer and autumn)	0213	30	0.35	0.35	12.15
*Cuscuta chinensis* Lam.	Convolvulaceae	Mulupabie	vegetable, vitamine & functional food	Whole plant, boiled in water (TC).	SC-CA-UA (all seasons)	0156				
*Cornus capitata* Wall.	Cornaceae	Laka	vitamine & functional food	Fruits, eaten raw (TC).	FO-CA-UA(summer and autumn)	0086	52	0.60	0.60	14.04
*Cyperus* sp.	Cyperaceae	Wongdanzi	forage	Aerial part, eaten raw or boiled in water (TC).	CA(spring, summer and autumn)					
*Dioscorea deltoidea* Wall. ex Griseb.	Dioscoreaceae	Rua ba; Luanba	carbohydrates	Tubers, dried and boiled in water (TC).	FO-CA-SC (autumn)	0094	7	0.08	0.08	4.73
*Dioscorea yunnanensis* Prain & Burkill	Dipsacaceae		vitamine & functional food	Roots, boiled in water (TC).	FO-SC-CA-UA (all seasons)	P1409				
*Diospyros lotus* L*.*	Ebenaceae	Tazhu	vitamine & functional food	Fruits, eaten raw (TC).	FO-CA-UA(summer and autumn)	P1417	3	0.03	0.03	1.01
*Elaeagnus umbellata* Thunb.	Elaeagnaceae		vegetable	Fruits, eaten raw (TC).	SC-UA (autumn)	0211				
*Hippophae rhamnoides* L.	Elaeagnaceae	Zhu	beverage	Fruits, fermented for sour taste (AB).	FO-SC (autumn)					
*Pyrola atropurpurea* Franch.	Ericaceae		forage	Aerial part, eaten raw or boiled in water (TC).	FO (spring, summer and autumn)					
*Vaccinium fragile* Franch.	Ericaceae	Anmiximi	vitamine & functional food	Fruits, eaten raw (AB).	FO-CA-UA(summer and autumn)	0021				
*Bauhinia* sp.	Fabaceae	Huangrekei	forage	Leaves, eaten raw or boiled in water (TC).	FO (spring, summer and autumn)	0142				
*Cassia* sp.	Fabaceae	Wujibaba	forage	Leaves, eaten raw or boiled in water for livestocks (TC).	FO (spring, summer and autumn)	0319				
*Lespedeza* sp.	Fabaceae	Fushibeibei	forage	Leaves, eaten raw or boiled in water (AB).	FO (spring, summer and autumn)	0100				
*Lespedeza thunbergii* subsp. *elliptica* (Benth. ex Maxim.) H. Ohashi	Fabaceae		forage	Leaves, eaten raw or boiled in water (AB).	FO(spring, summer, autumn)	0091				
*Medicago lupulina* L.	Fabaceae	Mosu	forage	Aerial part, eaten raw or boiled in water (TC).	CA(spring, summer and autumn)	0239				
*Piptanthus nepalensis* (Hook.) Sweet	Fabaceae	Murekei	forage	Leaves, eaten raw or boiled in water (TC).	FO(spring, summer and autumn)	0105				
*Trifolium repens* L.	Fabaceae		forage	Aerial part, eaten raw (TC).	CA(all seasons)	P1415				
*Quercus* sp.	Fagaceae	Laba	forage	Tender Leaves, eaten raw or boiled in water (TC).	FO (all seasons)	0098				
*Gentiana rigescens* Franch.	Gentianaceae	Yinini; Zii	vitamine & functional food	Whole plant, boiled in water (TC).	FO-SC (all seasons)	0326				
*Helwingia chinensis* Batalin	Helwingiaceae	Ninahagubii	vegetable	Tender leaves, fried (AB).	FO (spring and summer)	0215	4	0.05	0.05	0.90
*Hypericum forrestii* (Chitt.) N. Robson	Hypericaceae	Muwaniba	honey source plant	Flowers, sucked (AB).	SC-CA-UA (summer)	0243				
*Itea yunnanensis* Franch.	Iteaceae	Piejulu	forage	Tender leaves, eaten raw (TC).	FO-CA-UA(summer and autumn)	0077				
*Juglans cathayensis* Dode	Juglandaceae	Gudu	oil & fats	Seeds, dried and boiled in water (AB).	FO-SC-CA-UA (autumn and winter)	P1412	67	0.78	0.80	20.35
*Dracocephalum* sp.	Lamiaceae	Bingba	forage	Aerial part, eaten raw or boiled in water (AB).	FO (spring, summer and autumn)	0039				
*Elsholtzia strobilifera* (Benth.) Benth.	Lamiaceae		edible condiments	Seeds, dried, for seasoning (AB).	SC-CA-UA (autumn and winter)	0192				
*Mentha canadensis* L.	Lamiaceae	Angzhi	vegetable, edible condiments	Tender leaves and stems, fried, or cold and dressed with sauce (TC).	CA-UA (all seasons)	0012	43	0.50	0.50	169.31
*Origanum vulgare* L.	Lamiaceae	Kedu	edible condiments	Seeds and leaves, dried, for seasoning (AB).	SC-CA-UA (autumn and winter)	0058				
*Salvia* trijuga Diels	Lamiaceae		vitamine & functional food	Roots, boiled in water (TC).	FO-SC-UA (all seasons)	0119				
*Streptolirion volubile* Edgew.	Commelinaceae	Mailexu	forage	Aerial part, eaten raw (TC).	CA(spring, summer and autumn)	0030				
*Malva verticillata* L.	Malvaceae		carbohydrates	Tubers, dried and boiled in water (TC).	FO-CA-SC (autumn)	0152				
*Ficus sarmentosa* Buch.-Ham. ex Sm.	Moraceae	Kesulu	vitamine & functional food	Fruits, eaten raw (TC).	FO-CA-UA(summer and autumn)	0040	3	0.03	0.03	1.01
*Morus mongolica* (Bureau) C. K. Schneid.	Moraceae	Ciilu	vitamine & functional food	Fruits, eaten raw (TC).	FO-CA-UA(summer and autumn)	0132				
*Epipactis mairei* Schltr.	Orchidaceae	aba	forage	Aerial part, eaten raw or boiled in water (AB).	FO(spring, summer and autumn)	0026				
*Habenaria* sp.	Orchidaceae		vitamine & functional food	Tubers, boiled in water (AB).	FO (autumn)	0037				
*Oxalis acetosella* L.	Oxalidaceae	Tuolaibaba	forage	Aerial part, eaten raw or boiled in water (TC).	CA (spring, summer and autumn)	0313				
*Plantago asiatica* L.	Plantaginaceae	Umeiheizhou	vegetable, vitamine & functional food	Whole plant, boiled in water (TC).	SC-CA-UA (all seasons)	0049	64	0.74	0.78	144.00
*Avena fatua* L.	Poaceae	Wongdaba	carbohydrates forage	Seeds, dried, smashed and fried (TC). Whole plant for animal (TC).	CA (summer and autumn)	P1419				
*Catabrosa aquatica* (L.) P. Beauv.	Poaceae	Zii	forage	Aerial part, eaten raw or boiled in water (TC).	CA (spring, summer and autumn)	0256				
*Echinochloa crusgalli* (L.) P. Beauv.	Poaceae	Bai	carbohydrates	Seeds, dried (AB).	SC-CA-UA (summer, autumn and winter)	0146				
*Phyllostachys glauca* McClure	Poaceae	Zhusun	vegetable	Young shoots, fried (TC).	SC-CA-UA (spring and early summer)	0154	5	0.06	0.06	1.13
*Setaria viridis* (L.) P. Beauv.	Poaceae	Kucuzii	forage	Aerial part, eaten raw or boiled in water (TC).	CA (spring, summer and autumn)					
*Fagopyrum dibotrys* (D. Don) H.Hara	Polygonaceae	Saidiku	forage	Aerial part, eaten raw or boiled in water (TC).	CA (all seasons)	0015				
*Fagopyrum gracilipes* (Hemsl.) Dammer ex Diels	Polygonaceae	Niarlagulepo	forage	Aerial part, eaten raw or boiled in water (TC).	CA (spring, summer and autumn)	0141				
*Oxyria sinensis* Hemsl.	Polygonaceae	Huaji	vegetable, forage	Young shoots, eaten raw by people (AB). Leaves, eaten by animals (TC).	SC-CA-UA (spring, summer and autumn)	0176	10	0.12	0.23	15.19
*Polygonum capitatum* Buch.-Ham. ex D. Don	Polygonaceae	Niaorla	forage	Aerial part, eaten raw (TC).	CA (spring, summer and autumn)	0144				
*Polygonum paleaceum* Wall.	Polygonaceae	Yeku	vitamine & functional food	Roots, boiled in water (TC).	FO (all seasons)					
*Polygonum runcinatum* Buch.-Ham. ex D. Don	Polygonaceae	Lagasidi	vegetable	Leaves, fried (TC).	SC-CA-UA (spring, summer and autumn)	0237	6	0.07	0.07	4.68
*Rumex acetosa* L.	Polygonaceae	Lagasidi	vegetable	Young shoots, eaten raw (AB).	SC-CA-UA (spring, summer and autumn)	0236				
*Myrsine africana* L.	Primulaceae	Lagancii	vitamine & functional food	Fruits, eaten raw (AB).	FO-CA-UA(summer and autumn)	0076				
*Clematis armandii* Franch.	Ranunculaceae	Ehake	vegetable	Young shoots, fried (TC).	SC-CA-UA (spring)	0163				
*Clematis ranunculoides* Franch.	Ranunculaceae	Umeijuzi	forage	Aerial part, eaten raw or boiled in water (TC).	CA (spring, summer and autumn)	0046				
*Thalictrum aquilegiifolium* L*.* var. *sibiricum* Regel & Tiling	Ranunculaceae	Renuba	forage	Aerial part, eaten raw or boiled in water (TC).	CA(spring, summer and autumn)	0042				
*Ziziphus montana* W. W. Smith	Rhamnaceae	Cipa	vitamine & functional food	Fruits, eaten raw (TC).	FO-CA-UA(summer and autumn)	0349	17	0.20	0.20	5.74
*Amygdalus davidiana* (Carrière) de Vos ex Henry	Rosaceae	Buji,buka	vitamine & functional food	Fruits, eaten raw (TC).	FO-CA-UA(summer and autumn)	0217				
*Cerasus cerasoides* (Buch.-Ham. ex D. Don) S. Y. Sokolov	Rosaceae		vitamine & functional food	Fruits, eaten raw (TC).	FO-CA-UA(summer and autumn)	P1402				
*Docynia delavayi* (Franch.) C. K. Schneid.	Rosaceae	Sibu	vitamine & functional food	Fruits, eaten raw (TC).	FO-CA-UA(summer and autumn)		51	0.59	0.59	11.48
*Fragaria nilgerrensis* Schltdl. ex J. Gay	Rosaceae	Anmenbuzi; Alibuji; Ameibuji	vitamine & functional food	Fruits, eaten raw (TC).	FO-CA-UA(summer and autumn)	P1414				
*Fragaria vesca* L.	Rosaceae	Ameibuji	vitamine & functional food	Fruits, eaten raw (TC).	FO-CA-UA(summer and autumn)	0226	14	0.16	0.16	3.15
*Malus rockii* Rehder	Rosaceae		vitamine & functional food	Fruits, eaten raw (TC).	FO-CA-UA(summer and autumn)					
*Malus yunnanensis* (Franch.) C.K. Schneid.	Rosaceae	Lvba	vitamine & functional food	Fruits, eaten raw (TC).	FO-CA-UA(summer and autumn)	0210				
*Osteomeles schwerinae* C. K. Schneid.	Rosaceae	Dazhu	vitamine & functional food	Fruits, eaten raw (AB).	FO-CA-UA(summer and autumn)	0346	18	0.21	0.21	4.05
*Potentilla kleiniana* Wight & Arn.	Rosaceae		forage	Aerial part, eaten raw or boiled in water (TC).	CA (spring, summer and autumn)	0079				
*Prinsepia utilis* Royle	Rosaceae	Chuda	vitamine & functional food, beverage, oil & fats	Fruits, eaten raw (AB). Seeds, smashed and boiled in water for oil (CC). Leaves, for making functional tea (TC).	SC-CA-UA(spring, summer and autumn)	0159	45	0.52	0.57	177.69
*Pyracantha angustifolia* (Franch.) C. K. Schneid.	Rosaceae	Anmilaximi; Saigulu; Youlubuzhu	vitamine & functional food	Fruits, eaten raw (AB).	FO-CA-UA(summer and autumn)	0229	9	0.10	0.10	2.63
*Pyracantha fortuneana* (Maxim.) H. L. Li	Rosaceae	Abalugu	vitamine & functional food	Fruits, eaten raw (AB).	FO-CA-UA(summer and autumn)	0004	9	0.10	0.10	2.63
*Pyrus pashia* Buch.-Ham. ex D. Don	Rosaceae		vitamine & functional food	Fruits, eaten raw (TC).	FO-CA-UA(summer and autumn)	P1410				
*Rosa* sp.	Rosaceae	Haducii	vitamine & functional food	Fruits, eaten raw (TC).	FO-CA-UA(summer and autumn)	0128				
*Rubus biflorus* Buch.-Ham. ex Sm.	Rosaceae	Cipaaha	vitamine & functional food	Fruits, eaten raw (TC).	FO-CA-UA(summer and autumn)	0265	29	0.34	0.34	9.79
*Rubus* sp.	Rosaceae	Ciinaaha	vitamine & functional food	Fruits, eaten raw (TC).	FO-CA-UA(summer and autumn)	0172	29	0.34	0.34	9.79
*Sorbus hemsleyi* (C. K. Schneid.) Rehder	Rosaceae	Emaiji	vitamine & functional food	Fruits, eaten raw (TC).	FO-CA-UA(summer and autumn)	0274	48	0.56	0.56	135.00
*Sorbus hupehensis* C. K. Schneid.	Rosaceae	Yumaiji	vitamine & functional food	Fruits, eaten raw (TC). Dried and pounded to powder to cure high blood pressure (TC).	FO-CA-UA(summer and autumn)	0275	48	0.56	0.56	135.00
*Rubia membranacea* Diels	Rubiaceae		forage	Aerial part, eaten raw or boiled in water (TC).	CA(spring, summer and autumn)	0121				
*Zanthoxylum armatum* DC.	Rutaceae		edible condiments	Fruit shells, dried, for seasoning (TC).	FO-SC-CA (autumn)	0139				
*Zanthoxylum bungeanum* Maxim.	Rutaceae	Yehuajiao	edible condiments	Fruit shells, dried, for seasoning (TC).	FO-SC-CA (autumn)	0078	5	0.06	0.08	13.78
*Houttuynia cordata* Thunb.	Saururaceae	Arunaha; Azina	vitamine & functional food, vegetable	Tender leaves, stems and roots, fried, or cold and dressed with sauce (TC). Leaves, boiled in water (TC).	CA-UA (all seasons)	0044	74	0.86	1.21	2164.50
*Schisandra* sp.	Schisandraceae		beverage	Fruits, for making liqueur (TC)	FO (spring)					
*Debregeasia orientalis* C. J. Chen	Urticaceae	Pimi	vegetable	Young shoots and flower, eaten raw (AB).	SC-CA-UA (spring)	0143	12	0.14	0.14	8.10
*Verbena officinalis* L.	Verbenaceae		vegetable, vitamine & functional food	Whole plant, boiled in water (TC).	SC-CA-UA (all seasons)	0014	11	0.13	0.13	96.80
*Viola* sp.	Violaceae	Lagagudu; Lagaseimei	vitamine & functional food	Fruits, eaten raw (AB).	FO-CA-UA(summer and autumn)	0180	4	0.05	0.05	1.35
*Ampelopsis delavayana* Planch.	Vitaceae	Gaiha	vitamine & functional food	Fruits, eaten raw (TC).	FO-CA-UA (summer and autumn)	0084				
Gymnosperm										
*Pinus armandi* Franch.	Pinaceae	Situo	carbohydrates	Seeds, eaten raw (TC).	FO-SC (autumn)	0250	10	0.12	0.12	2.25
Fern										
*Equisetum hyemale* L.	Equisetaceae		forage	Aerial part, eaten raw or boiled in water (AB).	CA (spring, summer and autumn)					
*Adiantum* sp.	Pteridaceae		forage	Aerial part, eaten raw or boiled in water (TC).	FO (spring, summer and autumn)	0303				
*Pteridium aquilinum* var. *latiusculum* (Desv.) Underw. ex Heller.	Pteridaceae	Ade	vegetable	Leaves, fried (TC).	FO (spring and summer)	0222	38	0.44	0.44	51.3
*Pteridium revolutum* (Blume) Nakai	Pteridaceae	Angzhide	vegetable	Leaves, fried (TC).	FO (spring and summer)	0267	38	0.44	0.44	51.3
Mushroom										
*Auricularia* sp.	Auriculariaceae	Muer	vegetable	Fruit body, fried (TC).	FO (summer and autumn)	P1416				
*Boletus edulis* Bull.	Boletaceae	Chumugulu	vegetable	Fruit body, stewed, fried (TC).	FO (summer and autumn)	0342	8	0.09	0.09	4.80
*Tylopilus balloui* (Peck) Sing	Boletaceae	Niuganjun	vegetable	Fruit body, stewed, fried (TC).	FO (summer and autumn)					
*Tylopilus* sp.	Boletaceae	Bamu	vegetable	Fruit body, stewed, fried (TC).	FO (summer and autumn)		10	0.12	0.12	5.20
*Cantharellus cibarius* Fr.	Cantharellaceae	Jiyoujun	vegetable	Fruit body, stewed, fried (TC).	FO (summer and autumn)	P1405	10	0.12	0.12	6.00
*Cordyceps sobolifera* (Hill.) Berk. et Br.	Clavicipitaceae	Chongcao	vitamine & functional food	Fruit body, stewed (TC).	FO (summer)					
*Entoloma clypeatum* (L.) P. Kumm.	Entolomataceae	Yiwojun	vegetable	Fruit body, fried (TC).	FO (summer and autumn)	P1406				
*Gomphus* sp*.*	Gomphaceae	Labajun	vegetable	Fruit body, stewed, fried (TC).	FO (summer and autumn)					
*Hericium* sp.	Hericiaceae	Houtoujun	vegetable	Fruit body, stewed (TC).	FO (spring, summer and autumn)		5	0.06	0.06	2.60
*Laccaria laccata* (Scop.) Cooke	Hydnangiaceae	Tashimu	vegetable	Fruit body, stewed, fried (TC).	FO (summer and autumn)	0282				
*Hygrophorus* sp.	Hygrophoraceae	Huanglasan	vegetable	Fruit body, fried (TC).	FO (autumn)		3	0.03	0.03	1.80
*Engleromyces* sp.	Hypocreaceae	Zhujun	vegetable	Fruit body, dried and stewed (TC).	FO (summer)					
*Lentinula* sp.	Marasmiaceae	Zhemu	vegetable	Fruit body, fried (TC).	FO (summer and autumn)		6	0.07	0.07	3.60
*Morchella esculenta* Pers*.*	Morchellaceae	Aboduoluoluo	vegetable	Fruit body, stewed (TC).	FO (summer)					
*Coriolus versicolor* L.	Polyporaceae	Lingzhi	vitamine & functional food	Fruit body, stewed (TC).	FO(autumn)	P1401				
*Polyporus* sp.	Polyporaceae	Musi	vegetable	Fruit body, fried (AB).	FO-SC (spring and summer)	0278	3	0.03	0.03	1.80
*Ramaria rubri-attenuipes* R.H. Petersen & M. Zang	Ramariaceae	Saobajun	vegetable	Fruit body, stewed, fried (TC).	FO (summer)		12	0.14	0.14	6.24
*Lactaricus* sp. 1	Russulaceae	Wenzhishi	vegetable	Fruit body, stewed, fried (TC).	FO (spring and summer)	0338	14	0.16	0.16	8.40
*Lactaricus* sp. 2	Russulaceae	Baipajun	vegetable	Fruit body, stewed, fried (TC).	FO (spring and summer)	0341	17	0.20	0.20	10.20
*Lactaricus* sp. 3	Russulaceae	Minuka	vegetable	Fruit body, stewed, fried (TC).	FO (spring and summer)	0284				
*Lactarius hatsudake* Tanaka	Russulaceae	Tongbulu	vegetable	Fruit body, stewed, fried (TC).	FO (summer)	0285				
*Lactarius* sp. 4	Russulaceae	Angzhishi	vegetable	Fruit body, stewed, fried (TC).	FO (spring and summer)	0288				
*Lactarius* sp*. 5*	Russulaceae	Jucu	vegetable	Fruit body, stewed, fried (TC).	FO (spring and summer)	0281				
*Russula* sp. 1	Russulaceae	Kaca	vegetable	Fruit body, fried (TC).	FO (summer and autumn)	0286	9	0.10	0.10	5.40
*Russula* sp. 2	Russulaceae	Zhebu	vegetable	Fruit body, fried (TC).	FO (summer and autumn)	0340	9	0.10	0.10	5.40
*Russula* sp. 3	Russulaceae	Huotanjun	vegetable	Fruit body, fried (AB).	FO (summer)					
*Russula* sp. 4	Russulaceae	Azimenihu	vegetable	Fruit body, fried (TC).	FO (summer and autumn)	0283				
*Russula virescens* (schaeff . ex Zanted) Fr.	Russulaceae	Qingtoujun	vegetable	Fruit body, stewed, fried (TC).	FO (summer)		4	0.05	0.05	2.40
*Thelephora* sp.	Thelephoraceae	Ganbajun	vegetable	Fruit body, fried (TC).	FO (summer)					
*Tricholoma matsutake* Sing	Thelephoraceae	Songmaojun	vegetable	Fruit body, stewed, fried (TC).	FO (autumn)					
*Tricholoma* sp.	Thelephoraceae	Songmaojun	vegetable	Fruit body, stewed, fried (TC).	FO (autumn)					
*Tricholoma* sp.	Thelephoraceae	Yumu	vegetable	Fruit body, stewed, fried (TC).	FO (autumn)		19	0.22	0.22	41.04
*Lyophyllum fumosum* (Pers. : Fr.) P. D. Orton.	Tricholomataceae	Yiwojun	vegetable	Fruit body, fried (TC).	FO (summer and autumn)					
*Lyophyllum* sp.	Tricholomataceae	Menzher	vegetable	, stewed (TC).	FO (summer and autumn)	0280	31	0.36	0.36	22.32
*Marasmius* sp.	Tricholomataceae	Huangpijun	vegetable	Fruit body, fried (TC).	FO (summer)		3	0.03	0.03	1.80
*Termitomyces* sp. 1	Tricholomataceae	Mulu	vegetable	Fruit body, stewed, dried, dried and fried, salted (TC)	FO (summer and autumn)	P1403	36	0.42	0.42	116.64
*Termitomyces* sp. 2	Tricholomataceae	Umu	vegetable	Fruit body, stewed, dried, dried and fried, salted (TC)	FO (summer and autumn)	P1403				
Lichen										
*Lobaria retigera* Trevis.	Lobariaceae		vegetable	Whole plant, stewed, or cold and dressed with sauce (AB).	FO (all seasons)	0219	42	0.49	0.49	3.47
*Lobaria yunnanensis* Yoshim	Lobariaceae		vegetable	Whole plant, stewed, or cold and dressed with sauce (AB).	FO (all seasons)	0253	42	0.49	0.49	3.47
*Thamnolia vermicularia* (Sw.) Ach. Ex Schae	Thamnoliaceae		beverage	Whole plant, for making tea (TC).	RP (spring)					
Algae										
*Nostoc commune* Vaucher ex Bornet & Flahault	Nostocaceae	Baqi	vegetable	Aerial part, fried (TC).	AE (summer)	0279				
*Nostoc sphaeroids* Kutz	Nostocaceae	Bacai i e	vegetable	Aerial part, fried (TC).	FO (summer)	P1411				

Species in inventory are ordered from higher to lower plants, and they are arranged firstly by family taxa and then by genus taxa. Vernacular name of wild edibles are written using Chinese pinyin

The types of collecting habitats are based on the characterization proposed by Calabuig (2008)

*FC* frequency of citation, *CFSI* cultural food significance index, *CI* cultural importance index

^a^Prevalence of use: *AB* Abandoned, *CC* currently consumed, *TC* traditionally consumed

^b^Collecting habitat: FO Forests (oak woods, pine woods, etc.); SC Scrublands (*Pistacia*, etc.); AE Aquatic environments (streams, ditch, wet places, etc.); RP Rock places (rocky slopes, rocks, etc.); CA Cultivated areas (orchards, farmland, etc.); UA Urban and periurban areas (villages, roads etc.)

Voucher number with P means voucher photograph number, and the one without P means voucher specimen number

### Diversity of wild edibles

**Fig. 3:**
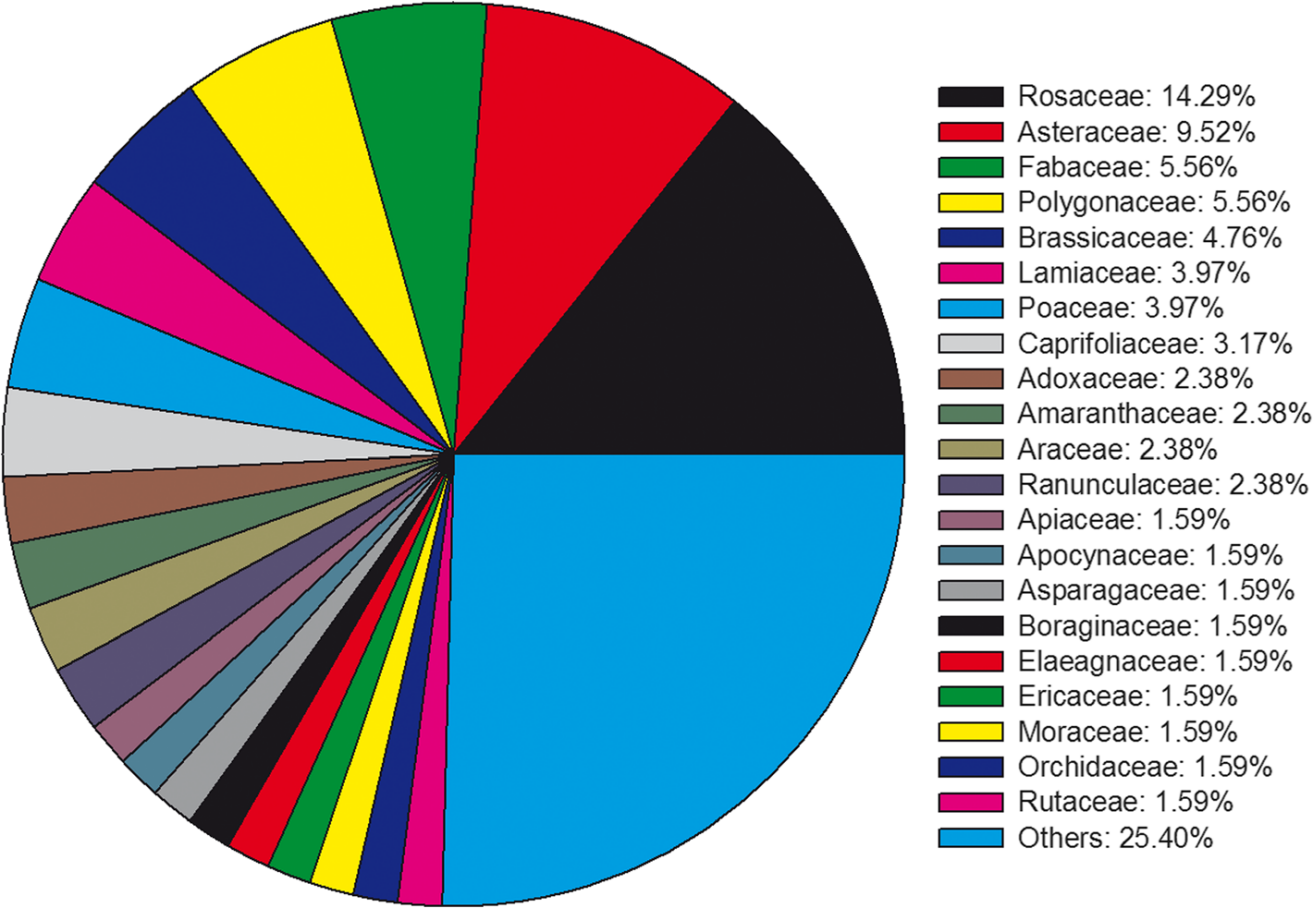
Family distribution of wild edible plant species of angiosperm category

Almost all major groups of wild plants in Baidi village have edible members that are reported to have been used by the indigenous Naxi people. Exceptions to the bryophytes, documented wild edibles include algae, lichen, fungi, fern, gymnosperm and angiosperm (Table 2). Most of the documented species were angiosperm with 126 species belonging to 53 families. Rosaceae was the biggest family with 18 wild edibles (Fig. 3), whereas 32 families contained only one edible plant species. Fungi was the second largest group containing 37 species representing 17 families. Gymnosperm included one species (one family), fern four species (two families), lichen three species (two families), and algae two species (one family). About one-sixth of 173 wild edibles were included in more than one food category, as listed in Table 3. As to the collecting habitats [29], most of these plants were collected from the wild populations nearby the village. It was also common that there was a small-scale cultivation of wild plants in the home gardens and all the space surrounding human habitations. Different plant parts were used as a source of food in Baidi village, but the most used parts were different depending on the purpose of the foods (such as forage and food medicines). Leaves, fruits, and the complete aerial parts were the mostly consumed by humans while the animals consumed leaves.

#### Wild vegetables

**Fig. 4:**
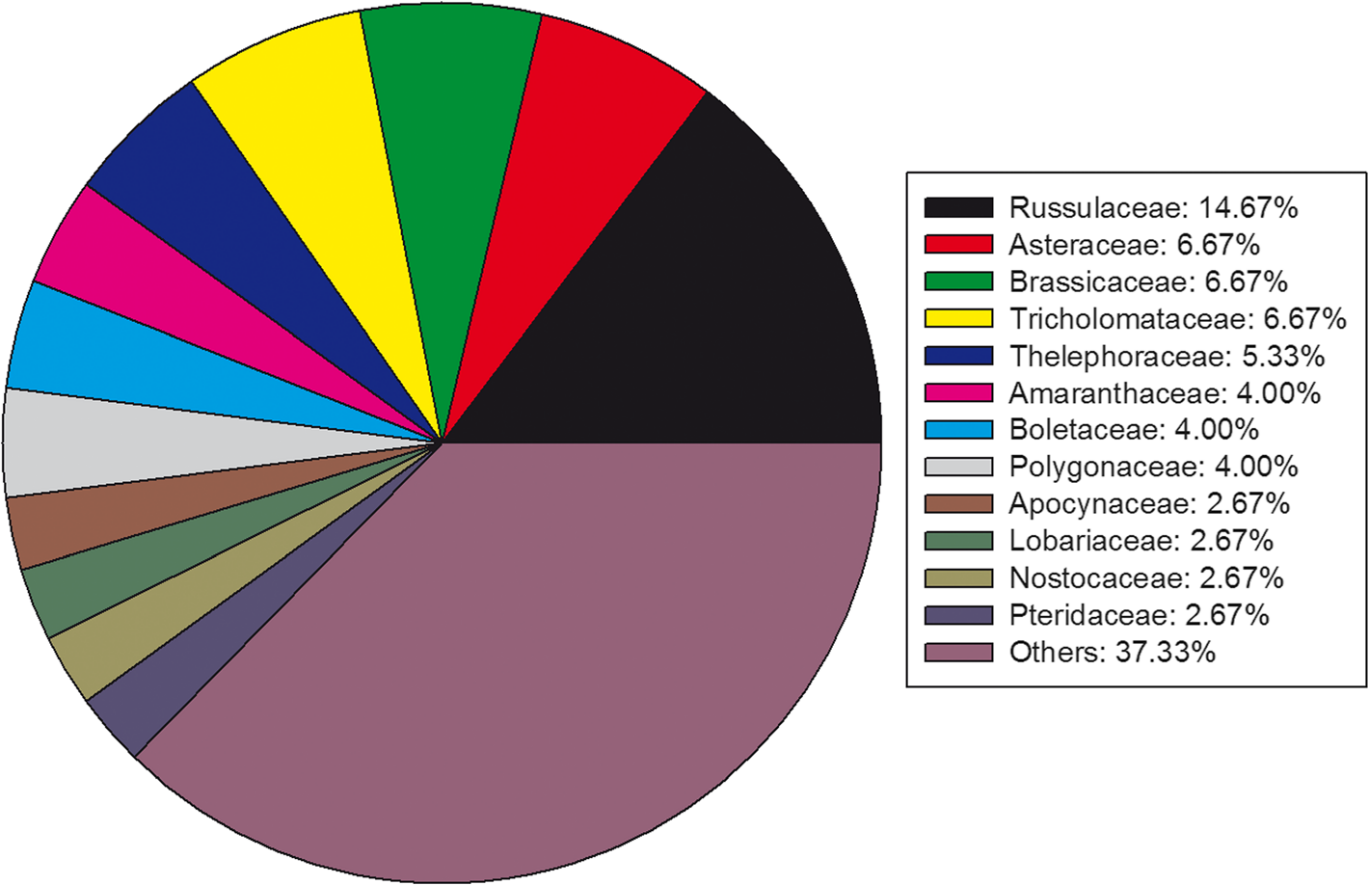
Percentage of wild vegetables in each family

This group was the biggest food category with 75 edible species belonging to 40 families. Russulaceae belonged to fungi had the highest number of species (11 species) eaten as vegetables (Fig. 4). Often wild vegetables were cooked in oil or fat or consumed in stews and soups. The most common procedure was to boil them first and then fried with garlic and chilies. The pork fat was common compared to vegetable oil for frying. The consumption of wild vegetable eaten raw was very rare.

The most frequently reported species were *Cardamine macrophylla* Willd., *C. tangutorum* O. E. Schulz., *Eutrema yunnanense* Franch. and *Houttuynia cordata* Thunb. All of these consumed after frying, except the last one, consumed as a salad with sauce. The first three species grow in the mountains, local people collected these species most often while grazing their cattle and horses during the spring and summer seasons. *Houttuynia cordata* grew wild in cropland and was collected by the local people when they finished their farm work. These four wild vegetables had been consumed for a long time, especially in the time of food shortages, later became the most popular vegetables in Baidi village. Wild gathered vegetables had different chemical composition and nutritional value from cultivated ones, according to Zeghichi [30]. Another two wild vegetables often used in the past, especially in time of food shortage, were the well-known *Lobaria retigera* Trevis. and *L. yunnanensis* Yoshim. (laolongpi is a vernacular name for both). These two plants are still consumed in other regions, like the Naxi in Lijiang city. These two species are proved to have high nutritional values such as antioxidant activity [31], but the Naxi in Baidi village abandoned this food tradition because of the unpleasant taste.

Most of the wild vegetables were defined as “bitter”, according to the Naxi, who related this to the concept of “healthy”. This kind of vegetable was considered “healthy” without any specification. According to Johns [32], such use had cultural significance related to the ingestion rather than taste.

Mushrooms also played a significant role in the local diet. Of 37 fungi species, most were eaten as vegetables and could be gathered during spring and fall. The mushrooms were consumed while fresh or after drying, and mostly grilled like meat. The harvesting of fungi for markets also had been one of main economic activities in the Baidi village. According to informants, *Boletus edulis* Bull., *Cantharellus cibarius* Fr. and *Entoloma clypeatum* (L.) P. Kumm., for example, were sold to Lijiang city, and to other provinces, such as Guangdong in Southern China. Women and children were the primary collectors of mushrooms. Mushrooms are the source of food in more than 80 countries worldwide, and their commercial harvesting is an important business in many countries, such as Turkey, the USA and Bhutan [33].

#### Vitamin and functional food

This food category included mainly wild fruits, which had a high content of vitamins and minerals, and food medicines consumed as both edible plants and medicinal plants. This group, with 60 species, was the second largest regarding the number of wild edibles cited.

Of the 30 wild edible fruits, Rosaceae was the largest family. Most of them did not have market value and sporadically gathered for household consumption, except *Malus pumila* Mill. and *Pyrus pashia* Buch.-Ham. ex D. Don. The most frequently eaten fruit was *Cornus capitata* Wall. for the Naxi. According to Johns [34], wild fruits are more fibrous and contain higher concentrations of vitamins and a greater diversity of secondary compounds compared to the cultivated species. Our study showed that many wild fruits were used as snacks, mainly by children in the past when cultivated fruits were not frequently available. They were probably a good source of vitamins and minerals but have become less important now, such as *Rubus biflorus* Buch.-Ham. ex Sm.

We documented 33 species belonging to food medicines. It is interesting that food medicines can also be wild vegetables and wild fruits. For example, *Houttuynia cordata* was delicious salad and antiphlogosis medicine. Similarly, *Sorbus hupehensis* C. K. Schneid. was tasty fruit and medicine to high blood pressure. Balick and Cox [35] explained aboriginal people do not make a clear distinction between edible and medicinal plants; we documented similar findings in the traditional practice of the Naxi in Baidi village. This kind of practices also exists in other Naxi villages in Shangri-La [15]. Moreover, some food preparations were taken exclusively for medicinal purpose, for example, *Habenaria* sp. fried with eggs was the most commonly used medicine for a cough [36, 37].

#### Carbohydrates and edible condiments

In the past, underground parts of some wild edibles such as *Dioscorea oppositifolia* that contain a high amount of starch used to be consumed, especially in the time of hardship. We documented six wild edibles used as the source of carbohydrates, out of that two were abandoned, and the remaining four were occasionally consumed. The main reason for the decrease in consumption was the diversity and abundance of cultivated crops in Baidi village. It was very common that wild edibles once frequently consumed in the past were now considered as weeds and rarely eaten. Such kind of change in perception has been reported from several places in Turkey, India and Brazil [38–40].

There were only six condiments from the wild source in the diet of Baidi village according to this study. The most often consumed species was *Zanthoxylum armatum* DC. The use of condiments not only enhances the flavor of certain dishes but also provides preservative and medicinal properties (anti-parasitic) [41].

#### Oil and fats, beverage and honey source plant

The Naxi in Baidi used total five wild edibles as a source of oil and fats, of which *Juglans mandshurica* Maxim. and *Cannabis sativa* L. were most commonly used. These two species were still widely used to make oil and fats. Similarly, *Prinsepia utilis* oil, rich in flavonoid and have been proved to have an anti-bacteria effect [42, 43], was also frequently used.

A total of four wild edibles recorded were used as the beverage. Fruits of *Schisandra* sp. were usually soaked in wine, which make the liquor medicinal [44, 45]. Leaves of the three species (*Hippophae rhamnoides*, *Prinsepia utilis* and *Thamnolia vermicularia*), were used to make vinegar and tea. Tea made of *Prinsepia utilis* has been proved to have significant immunosuppressive and antitumor activity [46, 47].

*Hypericum forrestii* (Chittenden) N. Robson was the only honey source plant. The local name for this species is “muwaniba”, which means it blooms during Dragon Boat Festival. This species with bright flowers attracts lots of bees during the flowering season, and local children have the habits of sucking its nectar for a sweet taste.

#### Forage

Altogether 40 wild species belong to 20 families were used as animal fodder in Baidi village. According to informants, they divided fodder plants into two groups: cropland group and mountain group based on the habitats. In mountain group, *Eutrema yunnanense* was the favorite fodder for the cattle. In cropland group, *Fagopyrum gracilipes* (Hemsl.) Dammer was often intentionally cultivated as animal fodder. Naxi women collected and carried those fodders from the cropland for stall feeding. The fodder plants also included *Oxyria sinensis* Hemsl. and *Cichorium intybus* L., the local people once consumed both of these during the food scarcity.

### Evaluating and selecting of wild edibles based on traditional wisdom

Twenty wild edibles were selected (Table 4) using four quantitative indices (FC, f, CI and CFSI). The ranks of some species based on different indices were different, indicating that different indices assigned particular importance of the various attributes, such as the multiplicity of uses and taste appreciation [27].

**Table 2:** Number of species and number of families in different plant categories

Plant categories	Number of families	Number of species
Angiosperm	53	126
Gymnospermae	1	1
Fern	2	4
Fungi	17	37
Lichen	2	3
Algae	1	2
Total	76	173

**Table 3:** Number of species in different food categories

Food categories	Number of species
Vegetable	75
Vitamin and functional food	60
Forage	40
Carbohydrates	6
Edible pigments	6
Oil and fats	5
Beverage	4
Honey source plant	1

**Table 4:** Evaluation of wild edibles (except forage category) of the Baidi village using four indices

Latin name	Vernacular name	Indices				Ranking			
		FC	f	CI	CFSI	FC	f	CI	CFSI
*Cardamine macrophylla* Willd.	You	76	0.88	0.94	205.20	1	1	2	3
*Cardamine tangutorum* O. E. Schulz	You	76	0.88	0.94	205.20	1	1	2	3
*Houttuynia cordata* Thunb.	Arunaha; Azina	74	0.86	1.21	2164.50	2	2	1	1
*Eutrema yunnanense* Franch.	Bei	73	0.85	0.85	65.70	3	3	4	13
*Taraxacum mongolicum* Hand.-Mazz.	Pugongying	70	0.81	0.85	157.50	4	4	4	6
*Cannabis sativa* L.	Samei	67	0.78	0.78	24.12	5	5	6	18
*Juglans cathayensis* Dode	Gudu	67	0.78	0.80	20.35	5	5	5	21
*Valeriana jatamansi* Jones	Matixiang	67	0.78	0.90	120.60	5	5	3	9
*Plantago asiatica* L.	Umeiheizhou	64	0.74	0.78	144.00	6	6	6	7
*Maianthemum japonicum* (A. Gray) La Frankie	Abu	53	0.62	0.62	55.65	7	7	7	14
*Cornus capitata* Wall.	Laka	52	0.60	0.60	14.04	8	8	8	24
*Docynia delavayi* (Franch.) C. K. Schneid.	Sibu	51	0.59	0.59	11.48	9	9	9	26
*Sorbus hemsleyi* (C. K. Schneid.) Rehder	Emaiji	48	0.56	0.56	135.00	10	10	11	8
*Sorbus hupehensis* C. K. Schneid.	Yumaiji	48	0.56	0.56	135.00	10	10	11	8
*Nasturtium officinale* R. Br.	Shuicai	45	0.52	0.52	206.72	11	11	12	2
*Prinsepia utilis* Royle	Chuda	45	0.52	0.57	177.69	11	11	10	4
*Mentha canadensis* L.	Angzhi	43	0.50	0.50	169.31	12	12	13	5
*Lobaria retigera* Trevis.	Laolongpi	42	0.49	0.49	3.47	13	13	14	38
*Lobaria yunnanensis* Yoshim	Laolongpi	42	0.49	0.49	3.47	13	13	14	38
*Allium* sp.	Gu	40	0.47	0.47	24.00	14	14	15	19

#### Food botanicals with high CI values

Wild edibles that had high CI values were *Houttuynia cordata* (1.21), *Cardamine macrophylla* (0.94), *C. tangutorum* (0.94), *Valeriana jatamansi* Jones (0.90) and *Eutrema yunnanense* (0.85). Whole plants of *Houttuynia cordata* and *Valeriana jatamansi* were consumed as functional food having a medicinal property, whereas the others were frequently eaten leafy vegetables.

Wild edibles with high CI values might have an interesting dietary constituent and needed further research. Also, a plant with a low CI value could be an important plant for a few people [27].

#### Food botanicals with high CFSI values

Wild edibles that had high CFSI values had different ranks from those with high CI values, and they were *Houttuynia cordata* (2164.50), *Nasturtium officinale* (206.72), *Cardamine macrophylla* (205.20), *Cardamine tangutorum* (205.20) and *Prinsepia utilis* (177.69). Three of them (*Houttuynia cordata*, *Cardamine tangutorum* and *Cardamine macrophylla*) were also in the front rank when assessed with CI values, but only *Houttuynia cordata* was positioned the same place when assessed with CFSI and CI values. *Eutrema yunnanense* growing on the high-elevation mountains ranked 13^th^ with CFSI index, attributed to its low availability index value, multifunctional food use index value and food-medicinal role index value. The local people consumed *Eutrema yunnanense* only as vegetables, and the collection was often time-consuming due to its mountain-grown habitat. While *Nasturtium officinale* and *Prinsepia utilis* were in the front position for their high availability index value, and food-medicinal-role index value respectively.

#### Traditional wisdom from the Naxi

**Fig. 5:**
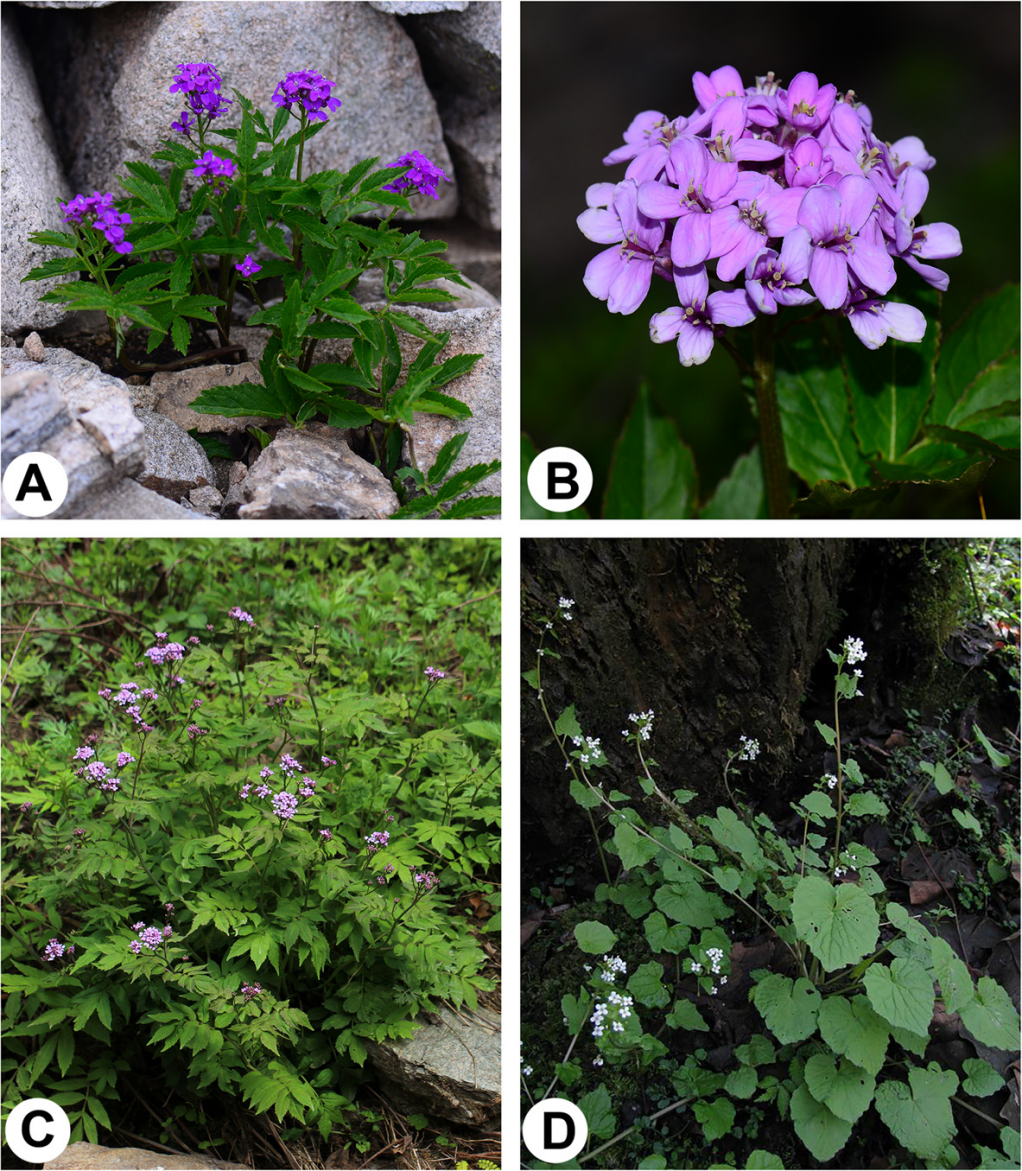
Three wild edible species with most promising exploitation prospects. **a** *Cardamine tangutorum*, whole plant; **b**. *C. tangutorum*, inflorescence; **c** *C. macrophylla*; **d**. *Eutrema yunnanense*. **a**-**c** photo by Renbin Zhu, (**d**) photo by Dahai Zhu

Our interview indicated a long history of consumption of *Cardamine macrophylla*, *C. tangutorum* and *Eutrema yunnanense* (Fig. 5). The results of quantitative indices showed that *Cardamine macrophylla*, *C. tangutorum,* and *Eutrema yunnanense* were in front positions. Hence, we selected these three species as most promising organic products.

Relevant literature studies show that high levels of vitamin C, minerals, fibers and protein have been reported in *Cardamine macrophylla* [48–52]. Also, low concentration of heavy metal has been found in this wild edible species. As its affinity, *C. tangutorum*, theoretically also had abundant nutrient components. Furthermore, other species of *Cardamine* are consumed as wild vegetables in Tanzania, India, Poland, United States and Slovakia [53–57]. *Eutrema wasabi* Maxim. is one of the raw materials of mustard. The species has proved to have anti-bacterial activity and flavor components [58, 59], and it has been developed as a condiment for many years by Lijiang Washabi Company *Eutrema yunnanense* widely consumed in Baidi village seems to be a potential vegetable. Additionally, as an affinity of *E. wasabi*, this species may have a similar chemical component with *E. wasabi*, and consequently use a substitute for *E. wasabi*.

Apart from the consumption in the rural area, the market of these wild edibles was expanded in the nearby city areas in the recent years. However, the scientific research regarding nutritional, phytochemical or phytopharmacological analysis was not conducted on the wild edibles recorded in Baidi village. In the context of increasing interest in the health potential foods, such as functional food and pharmafood, studies on wild edibles regarding the nutritional and medicinal qualities, and as potential alternative crops may be very useful [60]. The resurgence of the interest in the wild edibles was also consistent with a reappraisal of traditional cuisines, for example in European countries and with the general claim for ‘natural’ foods [61].

### Age, gender and knowledge dynamics

#### Age, gender, and traditional knowledge

**Fig. 6:**
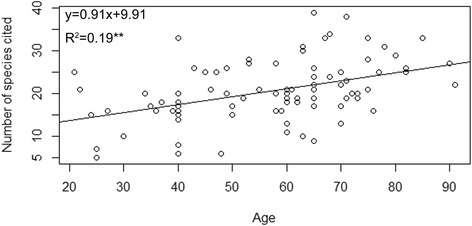
The relationship between informant age and number of species cited. (R^2^ = coefficient, and ** means *P value* is less than 0.01.)

All the informants in Baidi agreed that they consumed less number of wild edibles compared to the previous decades. Our results indicate the younger people almost could not identify, gather and process these species. Similarly, many middle-aged informants regarded the consumption of wild edibles as a symbol of poverty as they consumed these wild edibles during the time of scarcity. However, the gathering of wild edibles, such as *Cardamine macrophylla*, *C. tangutorum* and *Eutrema yunnanense* in the spring still represents a significant role in the daily diet. Overall, the number of wild edibles cited by informants increased with age according to our regression analysis (Fig. 6), even the correlation was weak (*P value* < 0.01, the coefficient = 0.19). Concurrent to our results, differences in the knowledge of wild food plants and wild edible fungi among different age groups is reported in two valleys of Shaanxi, central China [62]. However, decreasing knowledge trends in youngsters are common as in the case of other parts of the Himalayas [7]. A study in a Caribbean village finds that the older the people, the less they are affected by external influences [63]. In Baidi, many young people have migrated to other cities in Yunnan to search for employment and education in recent decades. According to our informants, such migration severely disrupted the transfer of local wild edibles knowledge between generations and led to the loss of TK.

The *t*-test results showed that there were no significant differences between females and males (*P value* = 0.361), even the number of species cited by women and men in different age groups fluctuated all the time. According to Pfeiffer and Butz [64], gender is a critical variable that influences local knowledge distribution, as it is highly correlated with other sociocultural factors including occupation, education, resource access, and social status and networks. Women tend to know more traditional knowledge [65, 66] because of the sociocultural factors mentioned above. Women are usually unemployed in the rural areas, dedicating themselves to the household and subsistence activities, and they combine this information with their cultural background as well as external knowledge to improve their subsistence [67]. Contrast to that in Baidi there was no clear-cut division of responsibilities for women and men, and they worked together in agriculture, leading to the matched food knowledge between men and women.

#### Knowledge transmission between Baidi Naxi and outsiders

According to our informants, two wild edibles, *Nasturtium officinale* and *Prinsepia utilis*, were currently consumed, and they represented different ways of knowledge acquisition. For *Nasturtium officinale*, local people learned the food use from the tourists from Guangdong province, and they spread the knowledge to near villages. For *Prinsepia utilis*, over the half of informants knew the fruits can be used to extract oil, but only one informant consumed the oil product. Some informants acquired the knowledge from their neighbors, relatives, and friends in nearby villages. There was one local market in Haba village (26 Km from Baidi village) for local people around to exchange goods, where also was a site for friends union and information dissemination. Since, the relatives play an important role as transmitters of knowledge and markets are significant sites for food knowledge transmission [68, 69], knowing who holds the traditional knowledge and ensuring the path to transmit it is meaningful ways to protect the knowledge.

The local name of the wild edibles was also helpful in recognizing the knowledge transmission pathways. The wild edibles that have a local Naxi name indicate prolonged consumption history, such as *Eutrema yunnanense*. The species that do not have local Naxi name may be introduced later, e.g. *Nasturtium officinale* (Xiyang cai, xiyang means western countries in Mandarin, cai means vegetable) and some fungi (muer, niuganjun etc.). It indicates that Naxi people may learn to use them from the Han Chinese as well as other minorities. Compared with another study in Shangri-La, many species utilized by the Naxi also are used by Tibetans [15], which may be one of the evidence of knowledge transmission.

## Conclusion

Baidi village is an excellent example of a rapidly changing village where local traditions compete with modern ways of life. Although many traditions have been lost in the past years, the Naxi in Baidi still preserves most of food traditions, especially the gathering of the wild species.

We documented 173 wild edible plant species representing 76 families 139 genera from our ethnobotanical survey. Some species were traditionally consumed as an important supplement to the diet, particularly during food shortages e.g. *Cardamine macrophylla*, *C. tangutorum* and *Eutrema yunnanense*, which also were potential wild food products with high nutritional value. The age factor significantly differred the traditional knowledge distribution, but there was no significant difference in knowledge between male and female informants. The traditional food knowledge of the Naxi in Baidi is dynamic, affected by social factors and communicated with the outsiders’ food knowledge. Overall, this study provides a deeper understanding of the Naxi traditional knowledge on wild edibles. The study suggests some wild edibles might have an interesting dietary constituent, which necessitates further investigation on the nutrition value as well as market opportunities. With scientific evidence on nutrition value and market opportunity, more people will be attracted toward the wild edibles that will help in addressing food security issues along with conservation of traditional knowledge of the aboriginal population.
